# Rigorous Bound on Hydrodynamic Diffusion for Chaotic Open Spin Chains

**DOI:** 10.1007/s10955-026-03658-3

**Published:** 2026-07-16

**Authors:** Dimitrios Ampelogiannis, Benjamin Doyon

**Affiliations:** Department of Mathematics, King’s College London, Strand, London, WC2R 2LS UK

**Keywords:** Diffusion, Open quantum systems, Spin chains, Hydrodynamics

## Abstract

The emergence of diffusion is one of the deepest physical phenomena observed in many-body interacting, chaotic systems. But establishing rigorously that correlation functions, say of the spin, expand diffusively, remains one of the most important problems of mathematical physics. We establish for the first time, with Lindbladian evolution, a lower bound on spin diffusion in chaotic, translation-invariant, nearest-neighbor open quantum spin-1/2 chain satisfying a local unitality condition and strong conservation of magnetization. The bound is strictly positive if and only if the local quantum jumps transport spin. Physically, the bound comes from the spreading effects of initial-state macroscopic fluctuations, a mechanism which occurs whenever spin is an interacting ballistic mode. Chaoticity means that the Hilbert space of extensive charges is spanned by magnetization; we expect this to be generic. Our main tool is the Green-Kubo formula, the mathematical technique of projection over quadratically extensive charges, and appropriate correlation decay bounds recently established. Because Lindbladian dynamics is not reversible, the Green-Kubo spin diffusion strength includes a contribution due to irreversibility, which we interpret as encoding the hydrodynamic entropy production that may occur in the forgotten environment. This, we show, vanishes for certain choices of interaction parameters, for which the Lindbladian dynamics becomes reversible. Our methods can be extended to finite or short ranges, higher spins, and other non-Hamiltonian systems such as quantum circuits. As we argue, according to the theory of nonlinear fluctuating hydrodynamics, we further expect these systems to display superdiffusion, and thus have *infinite* diffusivity; however this appears to still be beyond the reach of mathematical rigor.

## Introduction

Deriving the laws of hydrodynamics from the microscopic dynamics of many-body systems is a fundamental goal of statistical physics, and doing so rigorously forms an important part of Hilbert’s sixth problem. Of great importance is establishing the transport properties, and in particular diffusive transport, in a universal manner, as emerging from the microscopic dynamics. In this endeavor, a crucial step is to determine rigorous lower bounds on the strength of the diffusive spread of response functions, via the celebrated Green-Kubo formula for the Onsager matrix and Einstein’s relation connecting it to the diffusion parameters (see e.g. [1, 2]). The Onsager matrix is manifestly non-negative in parity-time-symmetric dynamics [2], leading to non-negative entropy production; however showing that it is strictly positive is a difficult problem. In quantum systems, this remains largely unsolved, with very few rigorous results. Focusing on spin chains, one such bound on spin diffusion at infinite temperature, derived by using special quadratically extensive charges, was obtained in [3], and a second bound by the curvature of the Drude weight in [4]. Both rely on the assumed absence of ballistic transport of spin and focus on certain integrable Hamiltonian models, where the charge is explicitly constructed. As far as we are aware, there exists no proof that the diffusion strength is strictly positive in chaotic quantum systems.

In general, there is a mechanism for diffusive effects, coming from large-scale fluctuations of the system’s emergent hydrodynamic modes. This happens if hydrodynamic modes exhibit *interacting ballistic transport* – more precisely, the eigenvalues of the flux Jacobian, which are the hydrodynamic velocities generalising the sound velocity in gases, are non-constant functions of the state. Indeed, in such cases, it is expected that fluctuations of the ballistic trajectories induced by initial-state macroscopic, thermodynamic fluctuations [5, 6], will lead to spreading that may contribute to diffusion [7, 8] and even anomalous effects [9–12]. A proof of this principle, which generalizes the ideas of [3], is found in [13] where the Onsager matrix is bounded rigorously by a projection formula onto quadratically extensive charges constructed from ballistic modes and representing initial-state fluctuations. This works in one-dimensional systems under the assumption of strong-enough clustering properties of correlations. This principle is important: although chaotic Hamiltonian chains do not admit ballistic transport of spin or energy, as follows from a general result of Kobayashi and Watanabe [14], as soon as the Hamiltonian structure is broken – such as in open chains (i.e. with incoherent processes) , or unitary quantum circuits (i.e. with discrete time) – interacting ballistic transport may arise. The distinction between Hamiltonian and non-Hamiltonian systems is crucial in the case of chaotic systems, as it is the Hamiltonian structure that forces the vanishing of persisent currents, as in [14], thus precluding the presence of ballistic transport in any chaotic Hamiltonian chain. Indeed, as we demonstrate here, there is a non-zero expectation value of the spin current, in a Gibbs thermal state, for a certain class of open spin chains, indicating ballistic transport. For quantum circuits one can also calculate a non-zero current, see [15, Equation 22] for an explicit example of a chaotic circuit with just one conserved charge that exhibits ballistic transport, hence it would also be worth exploring the present results in that setting. However, the bound in [13] is not explicitly non-zero, the presence of symmetries can in fact force it to be zero, and it relies on clustering assumptions that were until recently unproven.

In this paper, we construct a family of interacting, translation-invariant, nearest-neighbor spin-1/2 open (Lindbladian) infinite-length quantum chains, which conserve total magnetization, and for which we show an explicitly positive bound on the strength of linear-response spin diffusion. The models accounts for spin hopping, both coherently and incoherently, on nearest neighbors, along with phase decoherence effects. For the notion of diffusion to be meaningful in a Lindbladian system, it is natural to impose two properties which suggest that spin transport be of “hydrodynamic type". First, we require magnetization $M = \sum _{x\in {\mathbb {Z}}} \sigma _x^3$ to be *strongly conserved* (generating a strong symmetry [16]). This implies that we have a local spin conservation law. Second, we require the two-site local incoherent processes to satisfy a *local unitality condition* (a local version of unitality of the dynamics on finite spin chains, and related to quantum detailed balance [17–19]). Note that unitality has a simple physical meaning in the finite dimensional case, namely that the state cannot become less mixed over time, drawing an intuitive picture for the emergence of diffusion. We show that unitality makes Gibbs states with density matrix $e^{\mu M}$ stationary. Thus one may expect emergent hydrodynamics for the local conserved spin thanks to local thermalization. Transport is generically not reversible in Lindbladian systems, but we show that local unitality also implies the existence of a (different) time-reverse dynamics that is well defined on local observables in the infinite chain. The family of models we consider is the most general family of translation-invariant, nearest-neighbor spin-1/2 open chains solving these two requirements.

We note that Lindbladian systems can be seen as the quantum version of classical stochastic systems. There are many standard, rigorous mathematical techniques in order to establish the presence of non-zero diffusion in classical stochastic systems, see e.g. [20]. However, we emphasise that these standard techniques do not appear to be directly applicable to Lindbladian systems. The methods presented here are different, and, we believe, more widely applicable, including Lindbladian systems and, most likely, classical deterministic and stochastic systems as well.

For the family of Lindbladian chains described above, we show that the spin-spin Onsager coefficient is bounded by projection onto quadratic charges, and that the bound is strictly positive if and only if *incoherent transport processes generate a non-zero current*. When this happens, spin is an interacting ballistic mode as, we show, there is a spin current in Gibbs states that depends quadratically on the average local magnetization $\textsf {s}$, leading to a hydrodynamic velocity $v(\textsf {s})$ that depends on the $\textsf {s}$; the bound is proportional to $(v'(\textsf {s}))^2$ (Eq. (67) below). Physically, then, the bound accounts for the spreading effects of initial-state macroscopic fluctuations, the mechanism described above. We establish the bound by applying the result of [13], which we make fully rigorous by adapting the proof in order to use recent results on clustering of *n*-th order correlations [21]. Finally, we show that for certain choices of parameters, the Lindbladian evolution is in fact *reversible*, and the irreversibility diffusion strength vanishes. Thus, for this sub-family of models, we have established a strictly positive normal spin diffusion strength. The techniques will apply to any Lindbladian quantum system with strong spin conservation and local unitality, and we believe the results will generically hold.

By the theory of nonlinear fluctuating hydrodynamics [22], non-linear currents should give rise to the KPZ equation and superdiffusion. Rigorously establishing that the Onsager coefficient is infinite appears, however, to be yet inaccessible with current techniques.

### Overview of Results: Onsager Matrix and Explicit Bound

Because of the lack of time reversal (more precisely, parity-time-reversal) symmetry, constructing a manifestly non-negative quantity that measures diffusion requires special care. We construct a *normal diffusion strength* $\mathfrak {L}_\textrm{norm}(t)$ measuring the linear-response diffusive spread of forward spin propagation at time *t*, and an *irreversibility diffusion strength* $\mathfrak {L}_\textrm{irr}(t)$ measuring the diffusive spread of the mismatch, due to irreversibility, obtained by performing forward then backward evolution by time *t*:

1 $$\begin{aligned} \mathfrak {L}_\textrm{norm}(t)&= \frac{1}{t} \sum _{x\in {\mathbb {Z}}} (x^2-(v(\textsf {s})t)^2) \langle \tau _t^*(s(x)),s(0)\rangle ^c \end{aligned}$$

2 $$\begin{aligned} \mathfrak {L}_\textrm{irr}(t)&= \frac{1}{2t} \sum _{x\in {\mathbb {Z}}} x^2\langle \tau _{t}\tau ^*_t( s(x)),s(0)\rangle ^c. \end{aligned}$$

Here $\langle \cdot ,\cdot \rangle ^c$ is the connected correlation function in the Gibbs state $e^{\mu M}$ with average magnetization density $\textsf {s} = \textrm{Tr}e^{\mu M}\sigma ^3_0/\textrm{Tr}e^{\mu M}$; $A(x,t) = \tau _t^*(A(x))$ is the Heisenberg-picture forward Lindbladian time evolution of the local operator *A*(*x*); $\tau _t$ is the backward time evolution; $v(\textsf {s})$ is the hydrodynamic spin velocity in the Gibbs state; and $s(x)=\sigma _x^3$ is the spin operator at *x*. Note that, as a consequence of Eq. (53) derived in the main text, in the state with zero chemical potential ($\textsf {s}=0$), the hydrodynamic velocity vanishes v(0)=0, hence the formula for $\mathfrak {L}_\textrm{norm}(t)$ simplifies. Intuitively, the irreversibility diffusion strength encodes the entropy increase that may occur in the “forgotten" environment. Then we show, with full mathematical rigor and without extra assumption, in the general, explicit family of translation-invariant, nearest-neighbor spin-1/2 open chains with strong magnetization conservation and local unitality, Eqs. (2.1), (13), (14), that:

3 $$\begin{aligned} \mathfrak {L} = \liminf _{t\rightarrow \infty } \Big (\mathfrak {L}_\textrm{norm}(t) - \mathfrak {L}_\textrm{irr}(t)\Big ) \ge \frac{(\chi v'(\textsf {s}))^2}{8v_\textrm{LR}} \end{aligned}$$

where χ is the magnetic susceptibility and $v_\textrm{LR}$ is the Lieb-Robinson velocity. In the family of models considered, expressions for χ, $v'(\textsf {s})$ and $v_\textrm{LR}$ are given in (68), (69), and (16) with (17), respectively. We expect the bound in the form (3) to be universal for chaotic models with strong spin conservation and local unitality. Note that the limits $\lim _{t\rightarrow \infty } \mathfrak {L}_\textrm{norm}(t)$ and $\lim _{t\rightarrow \infty }\mathfrak {L}_\textrm{irr}(t)$ may in fact exist, but we have not proven this; in this case, the result is for the difference of these limits. The proof is obtained by naturally adapting the standard Green-Kubo formula, for spin diffusion, to Lindbladian evolution:

4 $$\begin{aligned} \mathfrak {L}&= \liminf _{T\rightarrow \infty } \frac{1}{T}\int _0^T\textrm{d}t \int _0^T\textrm{d}t'\,\sum _{x\in {\mathbb {Z}}} \langle j^-(x,t),j^-(0,t')\rangle ^c, \end{aligned}$$

5 $$\begin{aligned} j^-(x,t)&= j(x,t) - vs(x,t) . \end{aligned}$$

We use quadratic charge projections in order to lower-bound this quantity, and we show that it indeed decomposes into the normal and irreversible contributions as above.

The usefulness of the above results relies on the idea that there are no other extensive conserved quantities coupling to the spin current – as otherwise, additional ballistic spreading is expected to occur and the quantity $\mathfrak {L}$ is infinite. In its most general form, the Onsager matrix is

6 $$\begin{aligned} \mathfrak {L}_{mn}&= \liminf _{T\rightarrow \infty } \frac{1}{T}\int _0^T\textrm{d}t \int _0^T\textrm{d}t'\,\sum _{x\in {\mathbb {Z}}} \langle j_m^-(x,t),j_n^-(0,t')\rangle ^c, \end{aligned}$$

7 $$\begin{aligned} A^-(x,t)&= (1 - \mathbb {P}) A(x,t) \end{aligned}$$

where $j_m$ are the currents, and $\mathbb {P}$ is the projector onto the Hilbert space $\mathcal {H}_1$ of extensive conserved quantities [23] (we show that this Hilbert space exists for Lindbladian evolution with local unitality). As usual, this definition requires the knowledge of all extensive conserved quantities, which is difficult to establish in general, and (4) with (5) is obtained by assuming that the spin chain is chaotic, in the sense that $\mathcal {H}_1$ be one-dimensional, spanned by *M* only (there is only the spin current $j=j_1$). More precisely, we assume that *spin transport be chaotic*: the spin current projects only onto *M*. Proving chaoticity is a non-trivial task that is currently out of reach, as it requires one to establish the absence of other extensive charges [23–27]. In Hamiltonian systems there are exciting partial results, showing the absence of charges with local densities (a subset of extensive charges) in certain models [28–31]. In our understanding it is widely expected that generic interacting spin chains be chaotic.

## Set-Up

### Time Evolution

We consider a quantum spin chain with local spaces $M_2(\mathbb {C})$, spanned by the Pauli matrices and the identity matrix, on each site $x\in {\mathbb {Z}}$. On each finite $\Lambda \subset {\mathbb {Z}}$ we associate the algebra $\mathfrak {U}_{\Lambda }= \otimes _{i\in \Lambda } M_2({\mathbb {C}})$. The algebra of local observables is $\mathfrak {U}_\textrm{loc}= \cup _{\Lambda } \mathfrak {U}_{\Lambda }$ and consists of observables that are supported on a finite number of sites, and its Cauchy completion defines the quasi-local algebra of observables $\mathfrak {U}=\overline{ \cup _{\Lambda } \mathfrak {U}_{\Lambda }}$. For details on the definition of quasi-local $\hbox {C}^*$-algebras see [32, Chapter 6.2]. We rely on the $\hbox {C}^*$-algebra framework of statistical mechanics [32, 33] where the main object is the algebra of observables $\mathfrak {U}$, time evolution of the observables is defined by the Heisenberg picture and a state of the system is given by a positive linear functional of unit norm (corresponding to ensemble averages). We elaborate below.

We consider a homogeneous Lindbladian evolution where the generator of the dynamics is defined for each finite $\Lambda \subset {\mathbb {Z}}$ and contains both Hamiltonian nearest-neighbor interactions and dissipative two-site terms. The local Hamiltonian, for finite $\Lambda \subset {\mathbb {Z}}$, is

8 $$\begin{aligned} H_\Lambda = \sum _{x\in \Lambda } h(x), \quad h(x) \in \mathfrak {U}_{\{x,x+1\} } \subset \mathfrak {U}_\textrm{loc} . \end{aligned}$$

The Hamiltonian generates the reversible part of the dynamics. The local time evolution $\tau _{H}^{\Lambda }$ is defined as

9 $$\begin{aligned} \tau _t^{H_\Lambda } (A) = e^{itH_{\Lambda }} A e^{-itH_\Lambda } , \quad A \in \mathfrak {U} \end{aligned}$$

and its thermodynamic limit exists^1^ and is a strongly continuous automorphism which defines the reversible time evolution $\tau _t^H$ of the full system [32, Chapter 6]:

10 $$\begin{aligned} \tau _t^H (A) = \lim _{\Lambda \rightarrow {\mathbb {Z}}} e^{itH_\Lambda } Ae^{-itH_\Lambda } , \quad A \in \mathfrak {U}_\textrm{loc} \end{aligned}$$

which is extended to act on $\mathfrak {U}$ by continuity.

We note that boundary terms are not relevant in this framework, as all the quantities that we are interested in are ultimately calculated in the thermodynamic limit. This is one of the reasons why it is so fruitful to use the algebraic framework to extract hydrodynamic phenomena, as hydrodynamics is inherently a theory of the bulk.

The dissipative part is described by local quantum jump (or Lindblad) operators $L_i(x)\in \mathfrak {U}_{ \{x,x+1\}}$, $x\in {\mathbb {Z}}$ for *i* in some finite index set. The algebraic framework can be used for Lindbladian dynamics as well, and is quite powerful, as one is able to rigorously prove the existence of the thermodynamic limit of the dynamics and Lieb-Robinson bounds which are used below. We refer the reader to [34] for more details. On local observables $A\in \mathfrak {U}_{\Lambda }$ the Lindbladian $\mathcal {L}_{\Lambda }^*: \mathfrak {U}_{\Lambda } \rightarrow \mathfrak {U}_\textrm{loc}$ is a family of maps defined for all $\Lambda \subset {\mathbb {Z}}$ that acts as

$$\begin{aligned} \mathcal {L}_{\Lambda }^*(A) = \partial _t A(t) = i[H_{\Lambda },A] + \frac{1}{2}\sum _{x\in \Lambda ,\, i}\big (L_i(x)^\dagger [A,L_i(x)] + [L_i(x)^\dagger ,A]L_i(x)\big ). \end{aligned}$$

This is the standard form of the Lindbladian in the Heisenberg representation, but written in terms of commutators – this makes clear that its action is well defined on local operators, i.e. that $\mathcal {L}_{\Lambda }^*(A) \in \mathfrak {U}_\textrm{loc}$ for any $A\in \mathfrak {U}_\textrm{loc}$ and any $\Lambda \subset {\mathbb {Z}}$. Note, that the Lindbladian is also well-defined in the case $\Lambda ={\mathbb {Z}}$, in which case we will simply denote is as $\mathcal {L}^*$.

Time evolution of any $A\in \mathfrak {U}_\textrm{loc}$ is given by solving the initial value problem $\mathcal {L}_{\Lambda }^*(A) = \partial _t A(t)$ , A(0)=A and has a unique solution $(\tau ^*_t)_\Lambda A$, given by the flow $e^{t\mathcal {L}^*_\Lambda }$. The thermodynamic limit of the dynamics

11 $$\begin{aligned} \tau ^*_t(A) = \lim _{\Lambda \rightarrow {\mathbb {Z}}} e^{t\mathcal {L}^*_\Lambda } (A) \end{aligned}$$

exists for any $A \in \mathfrak {U}_\textrm{loc}$ and is a strongly continuous completely positive dynamical semigroup, and thus can be extended on $\mathfrak {U}$ [34].

We require strong spin conservation: the Hamiltonian density, and each quantum jump, are all required to preserve the total spin of the quantum chain,

12 $$\begin{aligned} {[}M,h(x)]= [M,L_{i}(x)] = 0,\quad M= \sum _{x\in {\mathbb {Z}}} \sigma ^3_x. \end{aligned}$$

Here and below, for any $A\in \mathfrak {U}_\textrm{loc}$ we write $[M,A(x)] = \lim _{\Lambda \rightarrow {\mathbb {Z}}} [M_\Lambda ,A(x)]$ with $M_\Lambda = \sum _{x\in \Lambda } \sigma ^3_x$. By spin conservation, the Hamiltonian density and quantum jump operators take the general forms

13 $$\begin{aligned} h(x) = \alpha \sigma ^+_x\sigma ^-_{x+1} + \bar{\alpha }\sigma ^-_x\sigma ^+_{x+1} + \beta \sigma ^3_{x}1\!\!1_{x+1} + \gamma \sigma ^3_x\sigma ^3_{x+1}, \end{aligned}$$

and

14 $$\begin{aligned} L_i(x) = a_i\sigma ^+_x\sigma ^-_{x+1} + b_i \sigma ^-_x\sigma ^+_{x+1} + c_i\sigma ^3_{x}1\!\!1_{x+1} + d_i 1\!\!1_{x}\sigma ^3_{x+1} + e_i\sigma ^3_x\sigma ^3_{x+1} \end{aligned}$$

where $\alpha \in {\mathbb {C}}$, $\beta ,\gamma \in {\mathbb {R}}$, $a_i,b_i,c_i,d_i,e_i \in {\mathbb {C}}$ are constants, which we will refer to as interaction parameters. Here, $\sigma _x^1$,$\sigma _x^2$,$\sigma _x^3 \in \mathfrak {U}_{ \{x \}}$ are the x, y and z Pauli matrices respectively acting on $\mathfrak {U}_{\{x \} }$ and $\sigma ^{\pm }= \frac{1}{2}(\sigma ^1 \pm i \sigma ^2)$. Note that for the Hamiltonian density it is not necessary to have the term $\sigma ^3_{x+1}$ as under $\sum _{x\in {\mathbb {Z}}}$ in *H* this is redundant; while for the Lindblad operator $L_i$ there is no redundancy. Observe that for $|a_i|>|b_i|$ the *i*th process leads to incoherent spin transport towards the left, while for $|a_i|<|b_i|$ it is towards the right; and the sum of terms for $c_i,d_i,e_i$ represent various types of incoherent local dephasing.

Time evolution for Hamiltonian systems satisfies the Lieb-Robinson bound [35], which can be extended to Lindbladian dynamics [34, 36], that yields a light-cone effect

15 $$\begin{aligned} \left\| [A(x,t),B] \right\| \le C_{A,B} e^{-\lambda (x-v_\textrm{LR}|t|)}. \end{aligned}$$

The Lieb-Robinson velocity $v_\textrm{LR}$ for (2.1) can be estimated by the results [34, 37]:

16 $$\begin{aligned} v_\textrm{LR} = 4e\zeta (2) V , \quad V=2 \left\| h(x)\right\| + 2 \sum _i \left\| L_i(x)\right\| \end{aligned}$$

where ζ is the Riemann function, and we can estimate for (13), (14):

17 $$\begin{aligned} V \le \tilde{V} = 2( 2|\alpha |+|\beta |+|\gamma |) + 2 \sum _i( |a_i|+|b_i|+|c_i|+|d_i|+|e_i|). \end{aligned}$$

Lieb-Robinson bounds are generally loose, and improvements can be made in the velocity estimates [38].

### Gibbs States and Inner Products

A state ω is defined as a positive, normalised to 1, linear functional of $\mathfrak {U}$ and ω(A) is interpreted as the ensemble average of the observable *A*. We consider the system to be in a Gibbs state $\omega _\mu$, for the total spin *M*, with chemical potential μ, defined as

18 $$\begin{aligned} \omega _\mu (A) = \lim _{\Lambda \rightarrow {\mathbb {Z}}} \frac{\textrm{Tr}_{\prod _{i\in \Lambda } {\mathbb {C}}^2}( e^{\mu M_\Lambda }A)}{\textrm{Tr}_{\prod _{i\in \Lambda } {\mathbb {C}}^2}(e^{\mu M_\Lambda })}. \end{aligned}$$

Below, when there is no ambiguity possible, we will write $\textrm{Tr}(\rho A)/\textrm{Tr}(\rho )$ to mean the limit $\Lambda \rightarrow {\mathbb {Z}}$ of $\textrm{Tr}_{\prod _{i\in \Lambda } {\mathbb {C}}^2}(\rho _\Lambda A)/\textrm{Tr}_{\prod _{i\in \Lambda } {\mathbb {C}}^2}(\rho _\Lambda )$. It is clear that the state $\omega _\mu$ satisfies a strong clustering condition: $\omega _\mu (AB) = \omega _\mu (A)\omega _\mu (B)$ for every $A\in \mathfrak {U}_\Lambda$, $B\in \mathfrak {U}_{\Lambda '}$ with $\Lambda \cap \Lambda '=\emptyset$.

Observables in $\mathfrak {U}$ can be organised in equivalence classes to construct Hilbert spaces with inner products which encode the relevant contributions at different hydrodynamic scales, see [13, 23, 27] where such Hilbert spaces are introduced. It is on these Hilbert spaces, and not on $\mathfrak {U}$, that certain important properties hold. From the state ω we define the sesquilinear forms $\langle \cdot , \cdot \rangle _0$ and $\langle \cdot , \cdot \rangle _1$ as

19 $$\begin{aligned} \langle A,B\rangle _0&:=\langle A^\dagger ,B\rangle ^c :=\omega ( A^{\dagger } B) - \omega (A^{\dagger })\omega (B)\end{aligned}$$

20 $$\begin{aligned} \langle A,B\rangle _1&:=\sum _{x \in {\mathbb {Z}}} \langle A(x),B\rangle _0, \quad A,B\in \mathfrak {U}_\textrm{loc}. \end{aligned}$$

We define the equivalence relations $A \sim _k A^{\prime }$ iff $\langle A- A^{\prime }, A- A^{\prime } \rangle _k=0$, k=0,1, and form the quotient spaces $\mathcal {V}_k=\mathfrak {U}_\textrm{loc}/ \sim _k$, which are pre-Hilbert spaces. Completing these spaces yields Hilbert spaces $\overline{\mathcal {V}_k} =\mathcal {H}_k$ where $\langle \cdot , \cdot \rangle _k$ is an inner product. $\mathcal {H}_0$ is simply related to the Gelfand-Naimark-Segal Hilbert space [33]. $\mathcal {H}_1$ is a Hilbert space whose elements we can think of as extensive quantities: note that any local observable is equivalent to its space translates. Hence we can think of the equivalence class of any local *A* as the formal sum ∑A(x) (the total *A* of the spin chain) and the inner-product carries information about the convergent connected correlations of such observables. These spaces are introduced and studied in [13, 23], where they are used to define hydrodynamic projections and extract diffusion from them.

The time evolution on $\mathfrak {U}$, $\tau ^*_t: \mathfrak {U} \rightarrow \mathfrak {U}$, generated by $\mathcal {L}^*$ as discussed around Equation (11), gives rise to time evolution operators on (the dense subspace of local elements of) the spaces $\mathcal {H}_k$.

This requires the Lieb-Robinson bound, adapting the proof of [23, Thm 5.11] and using the fact that $\tau _t^*$ is a contraction [34] of $\mathfrak {U}$: $\left\| \tau _t^*A\right\| \le \left\| A\right\|$. This is done by the local approximation $\sigma _n(\tau ^*_tA)$ of time evolved observables $\tau ^*_tA$, $A\in \mathfrak {U}_\textrm{loc}$, which is a direct consequence of the Lieb-Robinson bound [39]:

21 $$\begin{aligned} \left\| \sigma _n(\tau ^*_t{A})-\tau ^*_t{A}\right\| \le C_A e^{-\lambda (n- v_{LR}|t|)}, \quad n \in {\mathbb {N}}, t\in {\mathbb {R}}\end{aligned}$$

This approximation can be used to show that

22 $$\begin{aligned} \langle A(x,t),B(0)\rangle ^\textrm{c} \le C_{A}' e^{-\lambda (x- v_{LR}|t|)}, \quad t\in {\mathbb {R}}, x>v_{LR} |t| \end{aligned}$$

This follows simply in the case that the state satisfies the strong clustering condition mentioned above. It is a special case of a much more general result, whereby a clustering (in space) state combined with the Lieb-Robinson bound can be shown to be clustering for space-like translations, see [27, Appendix C].

We can also use the Lieb-Robinson local-approximation of time evolution to show $\lim _n \langle \sigma _n(\tau ^*_tA), B \rangle _k = \langle \tau ^*_tA,B \rangle _k$ exists for all $A,B \in \mathfrak {U}_\textrm{loc}$ and conclude that $\sigma _n(\tau ^*_tA)$ converges weakly in $\mathcal {H}_k$. This defines $\tau ^{\mathcal {H}_k}_t: \mathcal {V}_k \rightarrow \mathcal {H}_k$ by $\tau ^{\mathcal {H}_k}_t(A) = \textrm{w}-\lim \sigma _n(\tau ^*_tA)$. Below, by abuse of notation we will use the same symbols, $\tau _t$ and $\tau _t^*$, for time evolution on $\mathfrak {U}$, and on the Hilbert spaces $\mathcal {H}_k$, when there is no confusion possible.

### Local Unitality

We now impose what we refer to as *local unitality*.

In finite-dimentional settings, unitality is the condition that the Lindbladian operator acting on states, preserves the identity. Therefore, a natural notion of unitality of the dynamics that makes sense in the setting of spin chains on ${\mathbb {Z}}$ is that our Lindbladian evolution generated by $\mathcal {L}^*$ preserves the infinite-temperature state $\omega _0$. We will show that, in our homogeneous, local setting, this follows from local unitality, Eq. (25) below. This happens formally in much the same way that conservation of a global, extensive quantity follows from a local conservation law; likewise, associated to local unitality there is a “unitality current”. In the opposite direction, local dynamics and unitality strongly points towards the necessity of local unitality, although we are not aware of a general proof.

With strong spin conservation, local unitality in turn implies that Gibbs states $\omega _\mu$ are stationary, and that the natural “time-reverse process”, described by the exchange $L_i(x)\leftrightarrow L_i(x)^\dagger$, gives jump operators that are conjugate to those of the direct process with respect to inner product induced by the Gibbs states, Eq. (30) (thus this exchange is indeed naturally interpreted generating the time-reverse process). We note that these two properties seem to be related to notions of quantum detailed balance, here with respect to the Gibbs states $\omega _\mu$, discussed in [17–19]. But crucially, in the finite-dimensional case, unitality implies that the state *may only become more mixed* over time, see [40, Thm 1.3] and [41, 42]. Therefore, it is natural that our notion of local unitality imply that diffusion be strictly positive, our main result.

So, we have a clear set of conditions, local unitality and strong spin conservation, with a large family of explicit examples, for which our rigorous results hold. Note that here the presence of a strong symmetry is not strictly necessary – without it, the results in this subsection would hold for the infinite-temperature state $\omega _0$; but for the notion of diffusion, we need a non-trivial strong symmetry.

The condition of unitality in the infinite-dimensional case can simply be taken as

23 $$\begin{aligned} \omega _0(\mathcal {L}^*(A))=0 \end{aligned}$$

for all $A\in \mathfrak {U}_\textrm{loc}$. By cyclicity of $\omega _0$, this gives

24 $$\begin{aligned} \sum _{x\in {\mathbb {Z}}}\sum _{i} \omega _0( A[L_i(x),L_i(x)^\dagger ])=0. \end{aligned}$$

Note that $[L_i(x),L_i(x)^\dagger ]\in \mathfrak {U}_{\{x,x+1,x+2\}}$, and the sum over *x* of these must give 0 in (24). A natural way of solving this condition for every local *A* is using telescopic summation, and imposing *local unitality*, the following condition on the quantum jump operators:

25 $$\begin{aligned} \sum _i [L_{i}(x),L_{i}(x)^\dagger ] = o(x+1)-o(x) \end{aligned}$$

for some “unitality current" operator $o(x)\in \mathfrak {U}_\textrm{loc}$. Indeed, by telescopic summation, and clustering and homogeneity of the state $\omega _0$, (24) holds. Note that by strong spin conservation and locality of *o*(*x*), we have [M,o(x)]=0 for all *x*. The local unitality condition, along with strong spin conservation, guarantees that the Gibbs state $\omega _\mu$ is stationary. Indeed, for any $A\in \mathfrak {U}_\textrm{loc}$,

26 $$\begin{aligned} \omega _\mu (\mathcal {L}^*(A))&= \sum _{x\in {\mathbb {Z}}}\sum _{i}\omega _\mu (A[L_i(x),L_i(x)^\dagger ]) \nonumber \\&= \sum _{x\in {\mathbb {Z}}} \big (\omega _\mu (A o(x+1))-\omega _\mu (Ao(x))\big ) = 0 \end{aligned}$$

where in the first line we used the definition of $\mathcal {L}^*$ and strong spin conservation, and in the last we used again telescopic summation, and clustering and homogeneity of the state $\omega _\mu$. By a straightforward calculation we find that the local unitality condition is equivalent to

27 $$\begin{aligned} \sum _i a_i(\bar{d}_i - \bar{c}_i) = \sum _i \bar{b}_i(d_i-c_i), \end{aligned}$$

with $$o(x) = \sum _i\frac{|b_i|^2-|a_i|^2}{2} \sigma ^3_x$$.

As is clear from above, local unitality is the local version of the formal global relation

28 $$\begin{aligned} \sum _x\sum _i L_i(x)L_i^\dagger (x) = \sum _x\sum _i L_i^\dagger (x)L_i(x). \end{aligned}$$

Interpreting the exchange $L_i(x)\leftrightarrow L_i^\dagger (x)$ as time reversal for quantum jumps – a natural adaptation from classical stochastic processes – this has the basic understanding that “every process of energy transfer in one direction must also allow energy transfer in the opposite direction”. This is much like the classical detailed balance condition, but for quantum jump processes. (Note that for the Hamiltonian part, a natural quantum equivalent of detailed balance is often taken to be the KMS relation.) Our local unitality condition does not depend on the state – it is in fact a quantum version of the notion of detailed balance as applied to transition probabilities in stochastic processes, that is, the condition that there be at least one probability distribution that satisfies detailed balance for this transition probability. In this connection, the presence of a strong symmetry is the quantum version of the presence of a symmetry of the master equation of stochastic processes, which indeed often makes the stationary state non-unique (by breaking strict ergodicity). We also note that the derivation (26) makes it clear that any state which preserves *M* is stationary. Convex combinations of Gibbs states, often referred to as “mixtures” – are also stationary. This is again similar to the stochastic situation, where normalised linear combinations of probability distributions that satisfy detailed balance for a transition probability, also satisfy detailed balance for this transition probability. Gibbs states are, however, the physically relevant states in many-body physics, those that are at the basis of hydrodynamics.

As mentioned, local unitality has two important consequences:

#### Theorem 1

Take the context of subsections 2.1 and 2.1, and assume local unitality (25). That is, take (11), (13), (14) and assume (27). (1) The local Lindbladian defined by exchanging $L_i(x)\leftrightarrow L_i(x)^\dagger$ and changing the sign of the Hamiltonian,

29 $$\begin{aligned} \mathcal {L}(A) = -i[H,A] + \frac{1}{2}\sum _{x\in {\mathbb {Z}},\, i}\big (L_i(x) [A,L_i^\dagger (x)] + [L_i(x),A]L_i^\dagger (x)\big ) \end{aligned}$$

is conjugate to $\mathcal {L}^*$ with respect to both inner products above,

30 $$\begin{aligned} \langle \mathcal {L}^*(A),B\rangle _k = \langle A,\mathcal {L}(B)\rangle _k,\quad \langle \mathcal {L}(A),B\rangle _k = \langle A,\mathcal {L}^*(B)\rangle _k, \quad A,B\in \mathfrak {U}_\textrm{loc} \end{aligned}$$

and gives rise to a completely positive dynamical semigroup $\tau _t=\lim _{\Lambda \rightarrow {\mathbb {Z}}} e^{t\mathcal {L}_\Lambda }$ on $\mathfrak {U}$ with a Lieb-Robinson bound with (16). (2) Both $\tau _t^*$ and $\tau _t$ are contracting with respect to both inner products,

31 $$\begin{aligned} \langle \tau _t^*(A),\tau _t^*(A)\rangle _k \le \langle A,A\rangle _k,\quad \langle \tau _t(A),\tau _t(A)\rangle _k \le \langle A,A\rangle _k, \quad A\in \mathfrak {U}_\textrm{loc}, \end{aligned}$$

and thus can be extended to bounded operators forming semigroups on $\mathcal {H}_k$.

Explicitly, $\mathcal {L}$ is obtained from $\mathcal {L}^*$ by exchanging $b_i\leftrightarrow \bar{a}_i$ and $c_i,d_i,e_i\leftrightarrow \bar{c}_i, \bar{d}_i,\bar{e}_i$ in (14). We believe that the semigroups formed by $\tau _t$ and $\tau ^*_t$ on $\mathcal {H}_k$ can be shown to be strongly continuous, but this does not play any role here. As a consequence that $\tau _t^*$ is a bounded operator, the space of extensive conserved charges $\mathcal Q = \{q\in \mathcal {H}_1:\tau _t^*(q) = q\}$ [23], is a closed subspace, and the projection $\mathbb {P}:\mathcal {H}_1\rightarrow \mathcal Q$ in (6) makes sense. We interpret $\tau _t$ as the *backward time evolution* of the system, which is natural from (30); there is also a notion of extensive conserved quantities for backward time evolution. Note that $\mathcal {L}$ *does not* take the form the Lindbladian usually takes on density matrices; by contrast, the form above indeed makes sense on local operators in the infinite-volume setting. We show in Appendix Appendix B with certain choices of parameters, $\mathcal {L} = -\mathcal {L}^*$, in which case $\tau _t = \tau _{-t}^*$ and time evolution is unitary on $\mathcal {H}_k$: in this case the dynamics is reversible.

#### Proof

The form of $\mathcal {L}$ is the same as that of $\mathcal {L}^*$, exchanging $L_i(x)\leftrightarrow L_i(x)^\dagger$. Therefore, by the above discussion, it exists on $\mathfrak {U}_\textrm{loc}$, generates a strongly continuous dynamical semi-group on $\mathfrak {U}$ with a Lieb-Robinson bound on $\mathfrak {U}_\textrm{loc}$ and with the velocity (16), and this time evolution gives rise to a well defined evolution operator $\tau _t$ on the local elements of $\mathcal {H}_k$, k=0,1, and on their time translates by both $\tau _t$ and $\tau _t^*$.

What remains to be shown are Eqs. (30) and (31). Eq. (30) follows from evaluating connected correlation functions: using strong spin conservation (12) we have

32 $$\begin{aligned} \langle \mathcal {L}^*(A)^\dagger ,B\rangle ^c = \langle A^\dagger ,\mathcal {L}(B) \rangle ^c + \sum _{x\in {\mathbb {Z}},\,i}\langle A^\dagger ,\{B,[L_i(x),L_i(x)^\dagger ]\}\rangle ^c \end{aligned}$$

and using (25) the series vanishes by telescopic summation and clustering. The opposite order of $\mathcal {L},\mathcal {L}^*$ is obtained similarly.

For Eq. (31), this follows because local unitality essentially implies that the Lindbladian is unital, for which contraction properties are known [43]. However we are in the infinite-volume setting, so care must be taken. We provide a direct proof. We consider $\tau _t^*$, as the same proof holds for $\tau _t$.

It is sufficient to have the proof for $\langle \cdot ,\cdot \rangle _0$. Indeed, by translation invariance and the existence of the limit defining $\langle A,A\rangle _1$,

33 $$\begin{aligned} \langle A,A\rangle _1 = \lim _{\Lambda \rightarrow {\mathbb {Z}}} \frac{1}{|\Lambda |} \langle \sum _{x\in \Lambda }A(x),\sum _{x'\in \Lambda }A(x')\rangle _0. \end{aligned}$$

We consider, for any $A\in \mathfrak {U}_\textrm{loc}$,

34 $$\begin{aligned} \frac{d}{dt} \langle e^{t\mathcal {L}^*_\Lambda } (A),e^{t\mathcal {L}^*_\Lambda } (A)\rangle _0 = \omega _\mu (\mathcal {L}_\Lambda ^*(B^\dagger )B) + \omega _\mu (B^\dagger \mathcal {L}_\Lambda ^*(B)) \end{aligned}$$

with $B = e^{t\mathcal {L}^*_\Lambda } (A)$, and where differentiability holds because for Λ a finite set, we have finite matrices. The right-hand side is, by using strong magnetization conservation and the cyclic property of the trace

35 $$\begin{aligned} -\sum _{x,i} \omega _\mu \Big (L_i(x) B^\dagger BL_i(x)^\dagger + B^\dagger L_i(x)^\dagger L_i(x)B - L_i(x)^\dagger B^\dagger L_i(x) B - L_i(x) B^\dagger L_i(x)^\dagger B \Big ) \end{aligned}$$

where the sum on *x* is over $\Lambda = [-N,N]\cap {\mathbb {Z}}$. Now bound, using Cauchy-Schwartz inequality for the sesquilinear form $(C,D):=\omega _\mu (C^\dagger D)$ as $|(C,D)|\le \sqrt{(C,C)(D,D)}$ with $C = BL_i(x)$, $D = L_i(x)B$,

36 $$\begin{aligned} |\omega _\mu (L_i(x)^\dagger B^\dagger L_i(x)B)| \le \sqrt{\omega _\mu (L_i(x)^\dagger B^\dagger B L_i(x))\omega _\mu (B^\dagger L_i(x)^\dagger L_i(x)B)} \end{aligned}$$

and similarly,

37 $$\begin{aligned} |\omega _\mu (L_i(x) B^\dagger L_i(x)^\dagger B)| \le \sqrt{\omega _\mu (L_i(x) B^\dagger B L_i(x)^\dagger )\omega _\mu (B^\dagger L_i(x) L_i(x)^\dagger B)}. \end{aligned}$$

Writing $\omega _\mu (L_i(x)^\dagger L_i(x) B^\dagger B) = \frac{1}{2} \omega _\mu (L_i(x)L_i(x)^\dagger B^\dagger B) + \frac{1}{2} \omega _\mu (L_i(x)^\dagger L_i(x)B^\dagger B) + \frac{1}{2} \omega _\mu ([L_i(x)^\dagger ,L_i(x)]B^\dagger B)$, and using local unitality and translation-invariance of the state, we have, again by cyclic property of the trace and strong invariance,

38 $$\begin{aligned} \sum _{x,i} \omega _\mu (L_i(x) B^\dagger BL_i(x)^\dagger ) = \frac{1}{2} \sum _{x,i} \omega _\mu \Big (L_i(x) B^\dagger BL_i(x)^\dagger + L_i(x)^\dagger B^\dagger BL_i(x)\Big ) + \epsilon \end{aligned}$$

where $\epsilon = \epsilon (t) = \frac{1}{2}\langle o(N+1) - o(-N),B^\dagger B\rangle ^c$ (note that *B* depends on *t*). Similarly,

39 $$\begin{aligned} \sum _{x,i} \omega _\mu (B^\dagger L_i(x)^\dagger L_i(x) B) = \frac{1}{2} \sum _{x,i} \omega _\mu \Big (B^\dagger L_i(x) L_i(x)^\dagger B+ B^\dagger L_i(x)^\dagger L_i(x) B \Big ) + \bar{\epsilon }\end{aligned}$$

where $$\bar{\epsilon }= \bar{\epsilon }(t) = \frac{1}{2}\langle o(N+1) - o(-N),B B^\dagger \rangle ^c$$. With

40 $$\begin{aligned} r_1&=\sqrt{\omega _\mu (L_i(x)^\dagger B^\dagger B L_i(x))}\end{aligned}$$

41 $$\begin{aligned} r_2&=\sqrt{\omega _\mu (L_i(x)B^\dagger B L_i(x)^\dagger )}\end{aligned}$$

42 $$\begin{aligned} r_3&= \sqrt{\omega _\mu ( B^\dagger L_i(x)^\dagger L_i(x) B )}\end{aligned}$$

43 $$\begin{aligned} r_4&= \sqrt{\omega _\mu ( B^\dagger L_i(x)L_i(x)^\dagger B )} \end{aligned}$$

(keeping *x*, *i* implicit), we bound all terms that come with an overall positive sign in (35) – the 3rd and 4th terms – by their absolute values using (36) and (37), we use (38), (39) for the first two terms, and we bound the correction $-(\epsilon +\bar{\epsilon })$ by its absolute value, to obtain

44 $$\begin{aligned}  &   -\omega _\mu \Big (L_i(x) B^\dagger BL_i(x)^\dagger + B^\dagger L_i(x)^\dagger L_i(x)B - L_i(x)^\dagger B^\dagger L_i(x) B - L_i(x) B^\dagger L_i(x)^\dagger B \Big ) \nonumber \\  &   \qquad \le - \frac{1}{2} \sum _{x,i} \Big ( r_1^2 + r_2^2 + r_3^2 + r_4^2 - 2r_1r_3 - 2r_2r_4 \Big )+|\epsilon +\bar{\epsilon }| \nonumber \\  &   \qquad = - \frac{1}{2} \sum _{x,i} \Big ( (r_1-r_3)^2 + (r_2-r_4)^2 \Big )+|\epsilon +\bar{\epsilon }|\nonumber \\  &   \qquad \le |\epsilon +\bar{\epsilon }|. \end{aligned}$$

Therefore, we have

45 $$\begin{aligned} \frac{d}{dt} \langle e^{t\mathcal {L}^*_\Lambda } (A),e^{t\mathcal {L}^*_\Lambda } (A)\rangle _0 \le |\epsilon +\bar{\epsilon }|. \end{aligned}$$

By the Lieb-Robinson bound, for every t>0 there is $$\epsilon _N>0$$ such that $$|\epsilon (t')+\bar{\epsilon }(t')|\le \epsilon _N$$ uniformly on $t^{\prime }\in \left[0,t\right]$, and such that $$\lim _{N\rightarrow \infty } \epsilon _N = 0$$. Hence, integrating from t=0, we obtain

46 $$\begin{aligned} \langle e^{t\mathcal {L}^*_\Lambda } (A),e^{t\mathcal {L}^*_\Lambda } (A)\rangle _0 - \langle A,A\rangle _0 \le \epsilon _N t. \end{aligned}$$

Taking the limit $\Lambda \rightarrow {\mathbb {Z}}$ (implying $N\rightarrow \infty$ and therefore $\epsilon _N\rightarrow 0$), the result follows.□

## Lindbladian Onsager Coefficient

### Extensive Conserved Quantities and Chaoticity

In general, the relevant degrees of freedom for the hydrodynamic scale are the extensive conserved quantities, formally $Q_n = \sum _{x \in {\mathbb {Z}}}q_n(x)$, afforded by the microscopic dynamics, $\mathcal {L}^*(Q_n)=0$. With $q_n(x,t)$, $j_n(x,t)$ conserved densities and their currents, we have conservation laws $\partial _t q_n(x,t) + j_n(x,t)- j_n(x-1,t)=0$; it is those that control hydrodynamics. In fact, $Q_n$ as written above is formal, and does not exist in $\mathfrak {U}$. The correct construction of the space of extensive conserved quantities, which includes those with local or quasi-local densities ($q_n(x)\in \mathfrak {U}_\textrm{loc}$ or $\mathfrak {U}$) and possibly more, and the existence of the local currents, are studied in [23]. This space, Q, is in fact a Hilbert space, a closed subspace of $\mathcal {H}_1$ (because $\tau _t^*$ is bounded on $\mathcal {H}_1$), thus it is complete in the topology induced by $\mathcal {H}_1$. It is shown in [23] that, for Hamiltonian systems, it is this full Hilbert space Q that contributes to the Euler scale; it determines the exact Drude weight via a projection mechanism. Estimating the diffusive strength of any hydrodynamic mode requires, as per the Green-Kubo formula, subtracting its projection onto ballistic modes, and thus requires the full knowledge of this Hilbert space.

In Lindbladian systems, the closed space of extensive conserved quantities is likely to still play a crucial role, but the question of how is beyond the scope of this paper. Still, in the Green-Kubo formula (6) it is natural to project out time-invariant quantities. Systems with Lindbladian (2.1) may admit an infinity of conserved quantities, e.g. if integrable, which may happen also in the presence of incoherent processes [44, 45]; or a finite number, in which case we may characterise the system as being *chaotic*. In many integrable models, one knows how to write down the conserved charges iteratively, but proving that this set is complete, and no other charges exist, remains a conjecture. Likewise, proving that a system is chaotic remains an open problem. In our understanding, it is however expected that non-chaotic models are of measure 0 in the space of interactions.

We therefore assume that the only conserved extensive quantity of (2.1) is the magnetization $Q_1 = M= \sum _{x \in {\mathbb {Z}}} \sigma ^3_x$; that is $\mathcal Q = \textrm{span}(M)$. More precisely, in fact, we only need to assume that the spin current *j* (as an element of $\mathcal {H}_1$) projects onto *M*; physically, we assume that spin transport be chaotic. We emphasize that this assumption is only used in addressing the projection operator in the Green-Kubo formula (6). Once the formula is written for the projection onto magnetization, our lower bound result is rigorous. Note that *M* is also conserved by the backward time evolution $\tau _t$.

### Onsager Coefficient and its Decomposition

The spin current *j*(*x*) for spin density $s(x) = \sigma _x^3$ is defined by

47 $$\begin{aligned} \mathcal {L}^*(s(x)) + j(x)-j(x-1) = 0 \end{aligned}$$

and decomposes into a Hamiltonian (coherent) and Lindbladian (incoherent) parts, $j(x) = j^\textrm{H}(x) + j^\textrm{L}(x)$. Both can be straightforwardly evaluated, but we will only need the incoherent part (here we have used (27) to slightly simplify the expression):

48 $$\begin{aligned} j^\textrm{L}(x)&= \sum _i\Big ( 2|b_i|^2 P_{x}^+P_{x+1}^- - 2|a_i|^2 P_x^-P_{x+1}^+ \nonumber \\&\quad + \big (2a_i(\bar{d}_i-\bar{c}_i) + \bar{e}_i a_i - e_i \bar{b}_i\big )\sigma _x^+\sigma _{x+1}^- \nonumber \\&\quad + \big (2\bar{a}_i(d_i-c_i) + e_i \bar{a}_i - \bar{e}_i b_i\big )\sigma _x^-\sigma _{x+1}^+ \Big ) \end{aligned}$$

where $P^\pm _x = (1\pm \sigma _x^3)/2$ are local projections onto positive (up) and negative (down) spins. Our goal is to estimate the diffusive transport coefficient (4) for *j*(*x*).

With the chaoticity assumption, formula (6) simplifies to ($\mathfrak {L} = \mathfrak {L}_{11}$):

49 $$\begin{aligned} \mathfrak {L}&= \liminf _{T\rightarrow \infty } \frac{1}{T}\int _0^T\textrm{d}t \int _0^T\textrm{d}t'\,\sum _{x\in {\mathbb {Z}}} \langle j^-(x,t),j^-(0,t')\rangle ^c, \end{aligned}$$

50 $$\begin{aligned} j^-(x,t)&= j(x,t) - s(x,t) \chi ^{-1} \langle M,j(0,0)\rangle ^c \end{aligned}$$

where

51 $$\begin{aligned} \chi = \langle M,s(0,0)\rangle ^\textrm{c} \end{aligned}$$

is the magnetic susceptibility.

Note that $\sum _{x\in {\mathbb {Z}}} \langle j^-(x,t),j^-(0,t')\rangle ^c = \langle j^-(t),j^-(t')\rangle _1$ is the $\mathcal {H}_1$ inner product, that $\langle M,a(0,0)\rangle ^\textrm{c} = \langle s,a\rangle _1$, so that $s(x,t) \chi ^{-1} \langle M,j(0,0)\rangle ^c = (\mathbb {P}j)(x,t)$ is indeed the projection within the Hilbert space $\mathcal {H}_1$. The quantity of which we take the limit in (49) exists and is finite, by the Lieb-Robinson bound. Further, expression (49) is manifestly non-negative, $\mathfrak {L}\ge 0$, as, by averaging over positions using translation invariance, it is the expectation value of the square of a hermitian operator (see e.g. [26]). We take the infimum limit $\liminf _{T\rightarrow \infty }$ ([13, Eqs 39, 58]), as the limit itself, in (49), might not exist. The result may also diverge to infinity (indicating superdiffusion). By contrast to the Hamiltonian dynamics, because Lindbladian time evolution is not an automorphism of the operator algebra, $A(t) B(t) \ne \tau ^*_t(AB)$, even though the state is stationary, $\omega (\tau _t^*(A)) = \omega (A)$, the quantity $\langle j^-(x,t),j^-(0,t^{\prime })\rangle ^c$ is not a function of $t-t^{\prime }$.

What is the interpretation of formula (49)?

Technically diffusion occurs within a derivative expansion of the current, accounting for the principle that the system tends towards entropy maximization. In a long-wavelength state $\langle \cdots \rangle$, one writes phenomenologically $\langle j(x,t)\rangle = \textsf {j}(\langle s(x,t)\rangle ) -\frac{1}{2} \mathfrak {D}(\langle s(x,t)\rangle )\partial _x \langle s(x,t)\rangle + \ldots$ where $\textsf {j}(\textsf {s}) = \omega _\mu (j)$ for μ such that $\textsf {s} = \omega _\mu (s)$. It is a simple matter to evaluate the current and velocity: an explicit calculation of the trace is possible as it factorises into the individual sites, and we obtain

52 $$\begin{aligned} \textsf {j} = \omega _\mu (j^\textrm{L}(x)) = \sum _i \frac{|b_i|^2-|a_i|^2}{2\cosh ^2\mu }. \end{aligned}$$

As $\textsf {s} = \omega _\mu (\sigma _x^3) = \tanh \mu$, we can express the current as a function of the spin density, and therefore also the hydrodynamic velocity, obtaining

53 $$\begin{aligned} \textsf {j}(\textsf {s}) = \sum _i(|b_i|^2-|a_i|^2) \frac{1-\textsf {s}^2}{2},\quad { v(\textsf {s})= \frac{d \textsf {j}(\textsf {s})}{d \textsf {s}} =\sum _i(|a_i|^2-|b_i|^2)\, \textsf {s}.} \end{aligned}$$

Linear response arguments for the (phenomenologically justified) diffusive-scale hydrodynamic equation $\partial _t \textsf {s}(x,t) + v(\textsf {s}(x,t)) \partial _x \textsf {s}(x,t) = \frac{1}{2}\partial _x (\mathfrak {D}(\textsf {s}(x,t)) \partial _x \textsf {s}(x,t))$ then give an equation for the correlation $\langle s(x,t),s(0,0)\rangle ^c$, solved as a Gaussian centered around the hydrodynamic velocity:

54 $$\begin{aligned} \langle s(x,t),s(0,0)\rangle ^c \sim \frac{\chi }{\sqrt{2\pi \mathfrak {D}_\textrm{norm} t}}\, e^{-(x-vt)^2/(2\mathfrak {D}_\textrm{norm}t)}\qquad \text{(hydrodynamic } \text{ linear } \text{ response) } \end{aligned}$$

where we recall that $A(x,t) = \tau ^*_t(A(x))$ is the Heisenberg-picture time evolution of the local observable $A(x)\in \mathfrak {U}_\textrm{loc}$. Here we have renamed $\mathfrak {D}\rightarrow \mathfrak {D}_\textrm{norm}$ in order to distinguish it from the “irreversibility diffusion" discussed below; this is *normal* diffusion.

Recall that we have different forward and backward time evolutions. According to hydrodynamic intuition, both should lead to diffusion, thus there should be diffusion associated to irreversibility as well. Time evolution $\tau _t$ leads to the opposite average current $-\textsf {j}(\textsf {s})$, Eq. (53), thus the opposite hydrodynamic velocity. Evolving forward, then backward in time, and phenomenologically assuming diffusive extension around the end-point of the resulting ballistic trajectory, we thus expect, in analogy to hydrodynamic linear response,

55 $$\begin{aligned} \langle \tau _t \tau _t^*s(x),s(0,0)\rangle ^c \sim \frac{\chi }{\sqrt{4\pi \mathfrak {D}_\textrm{irr} t}}\, e^{-x^2/(4\mathfrak {D}_\textrm{irr}t)}\qquad \text{(hydrodynamic } \text{ linear } \text{ response). } \end{aligned}$$

This is *irreversibility* diffusion.

Using the relation

56 $$\begin{aligned} v(\textsf {s}) = \frac{d \textsf {j}}{d\mu } \Big / \frac{d\textsf {s}}{d\mu } = \chi ^{-1}\langle M,j(0,0)\rangle ^c \end{aligned}$$

we write from (50)

57 $$\begin{aligned} j^-(x,t) = j(x,t) - v s(x,t) \end{aligned}$$

and (4) (or (49)) with (5) follows. Using conservation laws, we obtain the physical meaning of (4). Following the steps of [2, App A] (the steps are made fully rigorous in App. Appendix C), we find that it decomposes into (the infimum limit of) the difference

58 $$\begin{aligned} \mathfrak {L} := \liminf _{t\rightarrow \infty } (\mathfrak {L}_\textrm{norm}(t)-\mathfrak {L}_\textrm{irr}(t)) \end{aligned}$$

of a normal linear-response diffusion strength

59 $$\begin{aligned} \mathfrak {L}_\textrm{norm}(t)&= \frac{1}{t} \sum _{x\in {\mathbb {Z}}} (x^2-(vt)^2) \langle s(x,t),s(0,0)\rangle ^c \end{aligned}$$

60 $$\begin{aligned}&= \frac{1}{t} \sum _{x\in {\mathbb {Z}}} (x^2 - (vt)^2) \frac{d}{d\mu }\frac{\textrm{Tr}\Big (e^{M + \mu s(0)} s(x,t)\Big )}{\textrm{Tr}e^{M + \mu s(0)} }\Bigg |_{\mu =0}, \end{aligned}$$

and an irreversibility diffusion strength

61 $$\begin{aligned} \mathfrak {L}_\textrm{irr}(t)&= \frac{1}{2t} \sum _{x\in {\mathbb {Z}}} x^2\langle \tau _{t}\tau ^*_t( s(x)),s(0,0)\rangle ^c \end{aligned}$$

62 $$\begin{aligned}&= \frac{1}{2t} \sum _{x\in {\mathbb {Z}}} x^2\frac{d}{d\mu }\frac{\textrm{Tr}\Big (e^{M + \mu s(0)} \tau _{t}\tau ^*_t(s(x))\Big )}{\textrm{Tr}e^{M + \mu s(0)} }\Bigg |_{\mu =0} \end{aligned}$$

the linear response of the spin evolved forward, and then backward, for the same time. If the correlation functions satisfy hydrodynamic diffusive equations, then (54) and (55) hold and we have Einstein relations for the limits $t\rightarrow \infty$:

63 $$\begin{aligned} \mathfrak {L}_\textrm{norm}(\infty ) = \mathfrak {D}_\textrm{norm}\chi ,\quad \mathfrak {L}_\textrm{irr}(\infty ) = \mathfrak {D}_\textrm{irr}\chi \quad \text{(hydrodynamic } \text{ linear } \text{ response) }, \end{aligned}$$

which provides the physical meaning of $\mathfrak {L}$. With the choices of parameters making the dynamics reversible, Appendix Appendix B, we have $\tau _t = \tau _{-t}^*$ and therefore $\mathfrak {L}_\textrm{irr}=0$.

## Main Result: Strictly Positive Spin Diffusion

To prove that the system exhibits at least diffusive transport, it suffices to find a lower, strictly positive, bound for $\mathfrak {L}$. In [13] a general bound is obtained in one-dimensional systems, under the assumption of clustering of *n*-th order connected correlations under space-time translations, which until recently remained without a proof. In finite range quantum spin chains the Gibbs states for nonzero temperatures are exponentially clustering [46], and the state $\omega _\mu$ is manifestly clustering because of ultra-locality of *M*. Combined with the Lieb-Robinson bound this implies space-like clustering for two-point connected correlations, [27, Appendix C]. Recently in [21] the authors established clustering of n-th order cumulants from two-point clustering, which is slightly different from the assumed clustering in [13]. In particular, applying [21, Theorem V.4] in the case of exponential clustering, we find that the *n*-th order connected correlations of $\omega _\mu$ satisfy:

64 $$\begin{aligned}  &   \langle A_1(x_1,t_1), A_2(x_2,t_2), \ldots ,A_n(x_n,t_n) \rangle ^c \le C_{A_1,\ldots ,A_n} e^{ -\nu z},\nonumber \\  &   \text {where } z=\max _i \{\min _{j, j\ne i} \{ {{\,\mathrm{\textrm{dist}}\,}}(A_i(x_i),A_j(x_j))\} \} \end{aligned}$$

for any $n\in {\mathbb {N}}$, $A_1,\ldots ,A_n \in \mathfrak {U}_{\textrm{loc}}$, $x_1,\ldots ,x_n\in {\mathbb {Z}}$ and $t_1,\ldots ,t_n \in [-v^{-1}z+1, v^{-1}z-1]$, where $C_{A_1,\ldots ,A_n},\nu >0$ constants^2^. This bound, in particular the decay with respect to the max-min distance *z*, captures the idea that n-th order connected correlations depend on an intermediate length-scale; whenever one observable is moved far away from all others, the correlation will decay no matter how close the rest of the observables are with each other. See also [47].

Consider the definition of the Onsager Coefficient, by equations 49 and 50. Under the clustering assumption Equation (64), we can apply Theorem 5.1 in [13] to rigorously obtain the following bound^3^:

65 $$\begin{aligned} \mathfrak {L} \ge \mathfrak {L}_\textrm{lower} := \frac{|\langle M ,M,j^-\rangle ^c|^2}{8v_\textrm{LR} (\langle M,s \rangle ^c)^2}. \end{aligned}$$

Here $M=\sum _x{\sigma _x^3}$ is the magnetisation, $s=\sigma ^3_0$ the spin density^4^, $v_{LR}$ the Lieb-Robinson velocity (16) and $j^-$ is the projection of the local spin currect (5). We provide an overview of the proof of 65 in Appendix Appendix A, which utilizes the two properties of Theorem 1.

If the three point connected correlation $\langle M,M,j^-\rangle ^c$ is non-zero, then the Onsager coefficient is strictly positive. We show the following:

### Theorem 2

Consider a quantum spin chain with Lindbladian evolution (2.1) with nearest neighbor Hamiltonian interaction (13) and Lindblad operators (14), the spin current (47), and the associated Onsager coefficient (4). It follows that for all interaction parameters $\alpha \in {\mathbb {C}}$, $\beta ,\gamma \in {\mathbb {R}}$, $a_i,b_i,c_i,d_i,e_i \in {\mathbb {C}}$ satisfying (27) and $\sum _i(|a_i|^2 - |b_i|^2)\ne 0$, the Onsager coefficient is strictly positive $\mathfrak {L}\ge \mathfrak {L}_\textrm{lower} > 0$.

### Proof

The bound (65) follows as a direct application of [13, Theorem 5.1]. We explain how the proof of [13, Theorem 5.1] applies to the Lindbladian case in Appendix Appendix A.

We proceed to calculate the bound (65) for (13), (14). Consider the connected correlation:

66 $$\begin{aligned} \langle M, M,j^-\rangle ^c = \langle M,M, j \rangle ^c - \chi ^{-1}\langle M, M, s\rangle ^c \langle M, j \rangle ^c. \end{aligned}$$

As $\langle M,a\rangle ^c = d\textsf {a}/d\mu$ and $\langle M,M,a\rangle ^c = d^2\textsf {a}/d\mu ^2$ for any observable *a* with average $\textsf {a}=\omega _{\mu }(a)$, changing variable $\mu \rightarrow \textsf {s}=\omega _{\mu }(s)$, the bound (65) can then be written in terms of the flux curvature $\textsf {j}''(\textsf {s}) = v'(\textsf {s})$ as67(this is valid in general in chaotic systems). Therefore, as

68 $$\begin{aligned} \chi = \frac{1}{(\cosh \mu )^{2}}>0, \end{aligned}$$

diffusion is strictly positive if and only if the flux curvature is non-zero (see also [13, Eq 1]). This above formula shows that if the hydrodynamic mode has state-dependent velocity $v(\textsf {s})$, then the above bound is strictly positive. With a state-dependent velocity, the hydrodynamic trajectory of the mode is affected by macroscopic fluctuations of the initial state, leading to diffusive effects. We interpret the bound above as arising from this diffusion mechanism.

The bound (65) can be calculated by elementary means. First, note that by the general result of [14] the Hamiltonian part $j^H$ of the spin current (47), vanishes in the Gibbs state $\omega _\mu (j^\textrm{H}(x)) = 0$. Thus our bound vanishes in generic Hamiltonian spin chain, both for energy and spin: the lack of ballistic transport does not allow the above diffusion mechanism to apply. However the incoherent processes lead to non-vanishing current and non-zero diffusion strength.

Indeed, we calculate the flux curvature

69 $$\begin{aligned} v'(\textsf {s}) = \textsf {j}''(\textsf {s}) = \sum _i(|a_i|^2-|b_i|^2) \end{aligned}$$

resulting in a lower bound for the spin-spin Onsager coefficient:

70 $$\begin{aligned} \mathfrak {L}_\textrm{lower} = \frac{ \big ( \sum _i(|a_i|^2-|b_i|^2) \big )^2 }{ 32e\zeta (2) \tilde{V} \cosh ^4(\mu )} \end{aligned}$$

where $\tilde{V}$ is defined in (17). We note that as soon as there is incoherent spin transport, $\sum _i|a_i|^2\ne \sum _i|b_i|^2$, spin is an interacting ballistic mode as according to (53) the hydrodynamic velocity $v = \textsf {j}'$ depends on the state, and we have a strictly positive diffusion bound. This shows Theorem 2. □

## Conclusion

We have shown that, in every translation-invariant chaotic nearest-neighbor open quantum spin-1/2 chain with strong spin conservation and local unitality, the spin-spin Onsager coefficient is strictly positive whenever there is incoherent spin transport (Eq. (70)). A chaotic spin chain is one for which the space of extensive conserved charges is one-dimensional (spanned by the magnetisation), therefore for which spin is phenomenologically expected to satisfy a single-component hydrodynamic equation. We expect that for almost every set of interaction parameters the spin chain be chaotic, thus admitting positive diffusion (Theorem 2). Rigorously establishing that chaoticity is generic remains a crucial open problem.

This is the first rigorous proof of strictly positive diffusion in chaotic spin chains, albeit for open chains, where the bound is for the difference of normal diffusion strength, and a diffusion strength associated to irreversibility of the dynamics. It is based on a general theorem of projection onto quadratically extensive charges, extending [3] and obtained by combining results of [13] and [21]. The bound encodes the mechanism by which diffusive effects arise because of fluctuations of hydrodynamic trajectories due to macroscopic, thermodynamic fluctuations in the initial state. The methods are easily extended to a finite number of explicit extensive charges, higher spins, and finite- or short-range interactions, and can also be applied to closed systems such as unitary quantum circuits and cellular automata, which can be shown to exhibit ballistic transport [15], and we expect the results still hold. Importantly, the methods do not work, for instance, for energy or spin transport in generic Hamiltonian quantum chains, because in Gibbs states there can be no persistent (ballistic) energy or spin current [14]: hydrodynamic velocities vanish, forbidding the above mechanism.

According to the theory of fluctuating hydrodynamics [22], the above mechanism should in fact lead to superdiffusion. Indeed, by expanding the Gibbs current around a spacetime stationary background as $\textsf {j}(\textsf {s}+\delta \textsf {s}) \approx \textsf {j} + \textsf {j}' \delta \textsf {s} + \textsf {j}'' \delta \textsf {s}^2/2$ and adding microscopic fluctuations, the hydrodynamic equation becomes $\partial _t \delta \textsf {s} + v\partial _x\delta \textsf {s}+ \textsf {j}'' \partial _x\delta \textsf {s}^2 = \frac{1}{2} \mathfrak {D} \partial _x^2\delta \textsf {s} + \partial _x \xi$ where ξ is an appropriately normalized delta-correlated white noise in space and time. This gives rise to the KPZ universality class if $\textsf {j}''\ne 0$, in which case $\langle s(x,t),s(0,0)\rangle ^c$ expands *superdiffusively* ($\mathfrak {L}=\infty$). According to (67), $\textsf {j}''\ne 0$ is exactly the condition for our diffusion lower bound to be strictly positive. Thus, although we have shown strict positivity of the Onsager coefficient, in fact, on phenomenological grounds, it is expected to be infinite. Rigorously showing $\mathfrak {L}=\infty$ in generic ballistic spin chains is another crucial open problem; results of [13] based on projections along with assumptions on the form of correlations may give a way forward.

## Data Availability

Data sharing not applicable to this article as no datasets were generated or analysed during the current study.

## Proof of the Onsager Coefficient Bound

Here, we give a proof of (65) relying on the results of [13]; the proof directly carries over to our case, after we resolve two technical difficulties.

In [13] hydrodynamic spaces are constructed from an initial algebra of observables, including the spaces $\mathcal {H}_k$ we defined here. These spaces capture finer scales of hydrodynamics, and their inner products are associated with the Drude Weight and Onsager matrix, capturing the respective transport properties. In these spaces, one defines extensive charges and in [13, Section 4], in particular Theorem 4.1, it is shown how the existence of such charges gives lower bounds on diffusion. More specifically, one defines a quadratically extensive charge by a sequence $Q= ( Q_n \in \mathcal {H}_1: n \in {\mathbb {N}})$ of elements of $\mathcal {H}_1$ with three properties on a dense subset $\mathcal {D}(Q) \subset \mathcal {H}_1$:

1. There exists γ>0 such that $\Vert Q_n\Vert _{\mathcal {H}_1}^2 \le \gamma n$ for all $n \in \mathbb {N}$ large enough.
2. The limit $Q''(a) := \lim _{n \rightarrow \infty } \langle Q_n, a \rangle _{1}$ exists for all $a \in \mathcal {D}(Q)$.
3. There exists k>0 such that $\lim _{n \rightarrow \infty } \sup _{s, t \in [-kn, kn]} |\langle Q_n, \tau _s a - \tau _t a \rangle _{1}| = 0$ for all $a \in \mathcal {D}(Q)$.

The definition of a quadratically extensive charge leads to a bound for the Onsager Coefficient, due to its relation to the inner products of the hydrodynamics spaces. This is done in Theorem 4.1 in [13], which is the abstract version of the bound we use here. We can explicitly construct these charges from the conserved quantities of a system, to obtain the explicit bound of Theorem 5.1 in [13]. In the general case, if $q_i \in \mathfrak {U}_\textrm{loc}$ is a local conserved quantity, then $Q_n = (1-\mathbb {P}) \sum _{-n}^n q_i(x) q_i$ (as an element of $\mathcal {H}_1$) is a quadratically extensive charge that can be used to bound the Onsager Coefficient [13, Equation 98]. In our case, this requires showing that $(M)_n :=(1-\mathbb {P}) \sum _{-n}^n \sigma ^3_x \sigma ^3_0$ (in the limit $n \rightarrow \infty$, as an element of $\mathcal {H}_1$) is a quadratically extensive conserved charge, i.e. it satisfies Points 1, 2, 3 above (see also [13, Section 4.2]). In the present set-up, we have two differences; we have a slightly different clustering property (64) and non-Hamiltonian evolution which does not act as an automorphism. We resolve these in the next two subsections. The rest of the proof follows exactly the same steps as in [13].

### Time Evolution

Here we consider the proof of Point 3 ($3^{\prime }$ in [13, Section 4.2]): there exists k>0

A1 $$\begin{aligned} \lim _n \sup _{t_1,t_2 \in [0,kn]} | \langle (M)_n, A(t_1)-A(t_2) \rangle _{1}|=0 \end{aligned}$$

for $A \in \mathfrak {U}_\textrm{loc}$ and $(M)_n = \sum _{-n}^n \sigma ^3_x \sigma ^3_0$. This is shown in [13] for Hamiltonian time evolution $\tau _t:\mathfrak {U} \xrightarrow {\sim } \mathfrak {U}$ and uses the property $\langle A(t), B\rangle _1 = \langle A, B(t)\rangle _1$ which holds by time invariance of the state ω and the fact that $\tau _t$ is an automorphism: $\tau _t^*(AB)= \tau _t(A)^* \tau _t(B)^*$. Then, (A1) is written as an integral of a time derivative and time evolution is moved on $(M)_n$, where we can use the conservation law [13, Eq. 121]. However, Lindbladian time evolution does not preserve the algebra, hence we are not generally able to move $\tau _t^*$ around in $\langle A(t), B\rangle _1$. However, we can take advantage of (30) from Theorem 1, to write:

A2 $$\begin{aligned} \langle (M)_n , \mathcal {L}^*A \rangle _1 = \langle \mathcal {L}(M)_n , A \rangle _1 \end{aligned}$$

For $\mathcal {L}(M)_n = \mathcal {L} \big ( \sum _{-n}^n \sigma ^3_x \sigma ^3_0 \big )$ we can use an “approximate Leibniz rule” satisfied by $\mathcal {L}$ on observables supported on just one site:

A3 $$\begin{aligned} \mathcal {L}( q(x) q(y)) = \mathcal {L}(q(x)) q(y) + q(x)\mathcal {L}(q(y)) + e(x,y) , \quad q(x) \in \mathfrak {U}_{ \{x\}},q(y) \in \mathfrak {U}_{ \{y\}} \end{aligned}$$

with the “error term" $e(x,y)\in \mathfrak {U}_\textrm{loc}$ is supported on a finite region containing *x* and *y*, vanishes for |x-y|≥2, and satisfies $\sum _{y=x-1}^{x+1} e(x,y) = \sum _{x=y-1}^{y+1} e(x,y)=0$^5^. Explicitly, we find e(x,x-1)=s(x)j(x-1)-j(x-1)s(x-1), e(x,x)=(j(x)-j(x-1))s(x)+s(x)(j(x)-j(x-1)), e(x,x+1)=j(x)s(x+1)-s(x)j(x), and one can check that it satisfies the sum rules by using the relation j(x)(s(x)+s(x+1))=0 (recall that $s(x) = \sigma _x^3$). Then, we have

A4 $$\begin{aligned} \sum _{x=-n}^n\langle \mathcal {L}(\sigma ^3_x) \sigma ^3_0 + \sigma ^3_x\mathcal {L}(\sigma ^3_0), A \rangle _1 \end{aligned}$$

and we can use the conservation laws $\mathcal {L}(\sigma ^3_x) = {j}(x) - {j}(x-1)$ (that is, the current with respect to backward time evolution is -j), use space invariance of $\langle \cdot ,\cdot \rangle _1$ and perfom the sum over *x* to get as in [13, Eq. 121]:

A5 $$\begin{aligned} \langle \mathcal {L}(M)_n, A \rangle _1 \big |_{t=0} = -\langle (M)_n, \mathcal {L}^*A \rangle _1 \big |_{t=0}= \langle {j}(-n) \sigma ^3_0 - {j}(n) \sigma ^3_0 -\sigma ^3_{-n} {j}_0 + \sigma ^3_{n} {j}_0 , A \rangle _1. \end{aligned}$$

The rest of the proof follows from [13, Eq. 122], where we are left with connected correlations of the form $\int _{t_1}^{t_2} \sum _{x\in {\mathbb {Z}}} \langle j(n,0) \sigma ^3(0,0) , A(x,t) \rangle ^c dt$, that are discussed below and vanish by clustering.

### Clustering

The proof of the Onsager matrix bound in [13, Theorem 5.1] is done under the assumption that for any $A_1,\ldots ,A_n \in \mathfrak {U}_\textrm{loc}$, any $x_1,\ldots , x_n \in {\mathbb {Z}}$, $t_1,\ldots , t_n \in {\mathbb {R}}$ there exists b,c>0 so that

A6 $$\begin{aligned} \langle A_1(x_1,t_1),\ldots , A_n(x_n,t_n) \rangle ^c \le f( \max _i \min _j \{ w_{ij} \}) \end{aligned}$$

where $w_{ij}=|x_i-x_j| - v_\textrm{LR} |t_i-t_j|$, $f(z)= c(b+z)^{-d}$. This is slightly different from the rigorously established clustering property (64) shown in [21], which instead holds for times $|t_i| \le v^{-1}z$, $z=\max \min \{{{\,\mathrm{\textrm{dist}}\,}}(A_i(x_i),A_j(x_j)) \}$ and bounds the connected correlations by a function of the max-min spatial distances and not the max-min space-time distances. However, the proof remains essentially unchanged. To see this, first note that (64) can be expressed in terms of the distances $|x_i-x_j|$ instead of ${{\,\mathrm{\textrm{dist}}\,}}(A_i(x_i),A_j(x_j))$ at the cost of a larger constant $C_{A_1,\ldots , A_n}$ that takes into account the diameter of ${{\,\mathrm{\textrm{supp}}\,}}(A_i)\cup {{\,\mathrm{\textrm{supp}}\,}}(A_j)$, see for example [48, Eq 121, Appendix C]. Note also that we only need to consider the last part of the proof of [13, Theorem 5.1], Equation (123), that requires use of clustering of connected correlators with observables at different times, as the rest of the proof has equal-time correlators and remains the same. Consider [13, Eq. 123]: let $A,B,C \in \mathfrak {U}_\textrm{loc}$ and the term $I_{s_1,s_2}(n):=\int _{s_1}^{s_2} dt \langle A(0,t),B(n,0)C\rangle _1$. We need to show that this vanishes when $s_1,s_2 \le n/(2v_\textrm{LR})$ as $n \rightarrow \infty$. It is sufficient to consider $s_1=0$, $s_2= nk$ for $k< 1/(2v_\textrm{LR})$. We have

A7 $$\begin{aligned}  &   |I_{s_1,s_2}(n)| \le \int _{s_1}^{s_2} dt \sum _{x \in {\mathbb {Z}}} | \langle A(x,t),B(n,0)C(0,0)\rangle ^c| \nonumber \\  &   \qquad = \int _{s_1}^{s_2} dt \int _{x \in {\mathbb {R}}} dx| \langle A(\lfloor x \rfloor ,t),B(n,0) C(0,0)\rangle ^c| \nonumber \\  &   \qquad = n^2 \int _{0}^{k} dt \int _{x \in {\mathbb {R}}} dx| \langle A(\lfloor nx \rfloor ,nt),B(n,0) C(0,0)\rangle ^c| \end{aligned}$$

Following [13, Eq 124], we split the space integral into two regions $R_1 = \{ |x - \frac{1}{2}| > \frac{3}{2}+kv_{{LR}} \}$ and $R_2 = \{ |x - \frac{1}{2}| \le \frac{3}{2}+kv_{{LR}} \}$, with t≤k. By [21, Lemma V.5] clustering is controlled by $z = \min ( \lfloor nx \rfloor -n, \lfloor nx \rfloor )$ if $t\le z/v_{LR}$, which holds in both $R_1,R_2$. Thus, the integrand is bounded exponentially and $I_{s_1,s_2}(n)$ vanishes at $n \rightarrow \infty$

## Reversible Lindbladian Spin Transport

We now show that, under the conditions

B8 $$\begin{aligned} \alpha = 0,\quad \sum _i(c_i-d_i)=0,\quad \sum _i(\bar{e}_i a_i - e_i\bar{b}_i)=0, \end{aligned}$$

which in particular imply local unitality (25), spin transport with Lindbladian $\mathcal {L}^*$ is reversible on $\mathcal {H}_k$, k=0,1:

B9 $$\begin{aligned} \tau ^*_t(s(x)) = \tau _{-t}(s(x)) \end{aligned}$$

for all $t\in {\mathbb {R}},\,x\in {\mathbb {Z}}$. In fact, this holds on the subspace $\mathcal {H}_{k,\mathrm spin}$ of $\mathcal {H}_k$ obtained from (the closure of) the space of local operators

B10 $$\begin{aligned} \mathfrak {U}_\textrm{spin} = \textrm{span}_\Lambda \Bigg (\prod _{y\in \Lambda }\sigma _y^3\Bigg )\ni s(x), \end{aligned}$$

that is $\tau ^*_t(A) = \tau _{-t}(A)$ for all $A\in \mathcal {H}_{k, \mathrm spin}$, and $\mathcal {L}^* = -\mathcal {L}$ on $\mathfrak {U}_\textrm{spin}$. In particular, both τ and $\tau ^*$ form *unitary groups* on $\mathcal {H}_{k,\mathrm spin}$ (which we conjecture can be shown to be strongly continuous). Hence, the setup of [23, Secs 4, 5] applies and projection theorems hold. Note that on $\mathfrak {U}_\textrm{spin}$, the Hamiltonian *H* with α=0 acts trivially – thus conditions (B8) and the restriction to these observables gives a model of pure quantum jumps.

For the proof, we only need to show that $\mathcal {L}^* = -\mathcal {L}$ on $\mathfrak {U}_\textrm{spin}$. We note that $\mathcal {L}^* = \sum _{x\in {\mathbb {Z}}} \mathcal {L}^*(x)$ where $\mathcal {L}^*(x)(A) = i[h(x),A] + \frac{1}{2}\sum _i L_i(x)^\dagger [A,L_i(x)] + [L_i(x)^\dagger ,A]L_i(x)$, and similarly for $\mathcal {L}$. Further, we have

B11 $$\begin{aligned}&\mathcal {L}^*(x)(\textbf{1}_x\textbf{1}_{x+1}) = 0,\ \mathcal {L}^*(x)(\sigma ^3_x \textbf{1}_{x+1}) = -j(x),\end{aligned}$$

B12 $$\begin{aligned}&\mathcal {L}^*(x)(\textbf{1}_x\sigma ^3_{x+1}) = j(x),\ \mathcal {L}^*(x)(\sigma ^3_x\sigma ^3_{x+1}) = 0. \end{aligned}$$

Under the condition (B8) we see that (1) the current $j(x)=j^\textrm{L}(x)$, Eq. (48) (recall that $j^\textrm{H}(x)=0$), lies in $\mathfrak {U}_\textrm{spin}$, and (2) $\mathcal {L}$, obtained from exchanging $b_i\leftrightarrow \bar{a}_i$ and $c_i,d_i,e_i\leftrightarrow \bar{c}_i, \bar{d}_i,\bar{e}_i$, gives the same results with j(x)↔-j(x). Therefore $\mathcal {L}^*(x)(o_xo_{x+1})=-\mathcal {L}(x)(o_xo_{x+1})\in \mathfrak {U}_\textrm{spin}$ for $o_xo_{x+1}\in \mathfrak {U}_\textrm{spin}$. Thus on any operator $A=\prod _{y\in {\mathbb {Z}}} o_y\in \mathfrak {U}_\textrm{spin}$ with $o_x\in \{\textbf{1}_x,\sigma ^3_x\}$ we have $\mathcal {L}^*(A) = \sum _{x\in {\mathbb {Z}}}\Big (\prod _{y\in {\mathbb {Z}}\setminus \{x,x+1\}}o_y\Big )\mathcal {L}^*(o_xo_{x+1}) = -\mathcal {L}(A)\in \mathfrak {U}_\textrm{spin}$, which completes the proof.

## Steps in the Derivation of (58)

We follow the steps of [2, App A], using discrete-space integration by parts $\sum _x x \partial _x j(x) = -\sum _x j(x)$, $\sum _x x^2 \partial _x j(x) = -\sum _x (1+2x)j(x)$, with $\partial _x j(x) = j(x)-j(x-1)$, and the Lieb-Robinson bound and clustering of correlation functions for boundary terms to vanish.

### 1. Definitions

Consider the spin density *s*(*x*, *t*) and spin current *j*(*x*, *t*), which satisfy the continuity equation:

C13 $$\begin{aligned} \partial _t s(x,t) + \partial _x j(x,t) = 0 \end{aligned}$$

with $\partial _x j(x) = j(x)-j(x-1)$ the discrete gradient. The projected current is defined as $j^-(x,t) = j(x,t) - v s(x,t)$, where the drift velocity *v* is given by $v = \chi ^{-1} \langle \sum _x j(x,0), s(0,0) \rangle ^c$, and χ is the magnetic susceptibility $\chi = \langle M,s(0,0)\rangle ^\textrm{c}$ . By construction, the projected current is orthogonal to the total charge: $\sum _x\langle j^-(x,t), s(0,0) \rangle ^c = 0$.

The Onsager coefficient $\mathfrak {L}$ is defined as in the main text:

C14 $$\begin{aligned} \mathfrak {L} = \liminf _{T\rightarrow \infty } \frac{1}{T} \int _0^T dt \int _0^T dt' \sum _{x \in \mathbb {Z}} \langle j^-(x,t), j^-(0,t') \rangle ^c \end{aligned}$$

and we take for $\langle \cdot ,\cdot \rangle ^\textrm{c}$ the connected correlation function (19) for the state $\omega _\mu$, Eq. (18). This state, we recall, has strong clustering properties: for every pair of local observables in $\mathfrak {U}_\textrm{loc}$, the equal-time connected correlation function vanishes for all large enough distances, and for finite time differences we have (22).

### Derivation

By clustering of correlations, the connected correlation $\langle j^-(x,t), j^-(0,t') \rangle ^c$ decays faster than 1/|*x*| at infinity. We apply IBP to the sum over *x*, where the boundary term vanishes:

C15 $$\begin{aligned} \sum _{x \in \mathbb {Z}} \langle j^-(x,t), j^-(0,t') \rangle ^c&= [x \langle j^-(x,t), j^-(0,t') \rangle ^c]_{-\infty }^{\infty } - \sum _{x \in \mathbb {Z}} x \partial _x \langle j^-(x,t), j^-(0,t') \rangle ^c \nonumber \\&= - \sum _{x \in \mathbb {Z}} x \partial _x \langle j^-(x,t), j^-(0,t') \rangle ^c. \end{aligned}$$

Using the continuity equation we have $\partial _x j^-(x,t) = -(\partial _t + v \partial _x) s(x,t)$. Substituting this into the previous expression:

C16 $$\begin{aligned} \sum _x \langle j^-(x,t), j^-(0,t') \rangle ^c = \sum _x x \partial _t \langle s(x,t), j^-(0,t') \rangle ^c + v \sum _x x \partial _x \langle s(x,t), j^-(0,t') \rangle ^c. \end{aligned}$$

Applying IBP to the second term yields $-v \sum _x \langle s(x,t), j^-(0,t') \rangle ^c = -v \langle M, j^-(0,t') \rangle ^c$. Since $j^-$ is orthogonal to the total spin *M*, this term is zero.

Integrating over t∈[0,T] using the Fundamental Theorem of Calculus:

C17 $$\begin{aligned} \int _0^T dt \sum _x \langle j^-(x,t), j^-(0,t') \rangle ^c = \sum _x x \langle (s(x,T) - s(x,0)), j^-(0,t') \rangle ^c. \end{aligned}$$

Using translation invariance we shift the index x→-y:

C18 $$\begin{aligned} \sum _x x \langle \Delta s(x), j^-(0,t') \rangle ^c = - \sum _y y \langle \Delta s(0), j^-(y,t') \rangle ^c \end{aligned}$$

where Δs(x)=s(x,T)-s(x,0). Applying a second spatial IBP and using again the fact that boundary terms vanish gives the terms $(y^2/2) \partial _y j(y)$ and (1/2)j(y). For the second, we use translation invariance and the fact that *M* is conserved giving a vanishing contribution. So we get

C19 $$\begin{aligned} \int _0^T dt \sum _x \langle j^-(x,t), j^-(0,t') \rangle ^c = \sum _y \frac{y^2}{2} \partial _y \langle \Delta s(0), j^-(y,t') \rangle ^c. \end{aligned}$$

Substitute $$\partial _y j^-(y,t') = -(\partial _{t'} + v \partial _y) s(y,t')$$ and integrate over $t^{\prime }\in \left[0,T\right]$:

C20 $$\begin{aligned} \mathfrak {L} = \liminf _{T \rightarrow \infty } \frac{1}{T} \sum _y \int _0^T dt' \left[ -\frac{y^2}{2} \partial _{t'} \langle \Delta s(0), s(y,t') \rangle ^c - \frac{v y^2}{2} \partial _y \langle \Delta s(0), s(y,t') \rangle ^c \right] . \end{aligned}$$

Integrating the first term and using IBP on the second (yielding $+ vy \langle \Delta s(0), s(y,t') \rangle ^c$):

C21 $$\begin{aligned} \mathfrak {L} = \liminf _{T \rightarrow \infty } \frac{1}{T} \sum _y \left( -\frac{y^2}{2} \langle s(0,T) - s(0,0), s(y,T) - s(y,0) \rangle ^c + v y \int _0^T dt' \langle \Delta s(0), s(y,t') \rangle ^c \right) . \end{aligned}$$

We use again the conservation law $\partial _y j^-(y,t) = -(\partial _{t} + v \partial _y) s(y,t)$, translation invariance, IBP and $\langle j^-(0,t),M\rangle ^\textrm{c}=0$ to write

C22 $$\begin{aligned} \sum _y y \langle \Delta s(0), s(y,t') \rangle ^c = -\sum _y y\int _0^T dt\,\partial _t \langle s(y,t), s(0,t') \rangle ^c = -v T \langle M,s(0,t')\rangle ^\textrm{c} \end{aligned}$$

and therefore, by the fact that *M* is conserved both under $\tau _t^*$ and $\tau _t$,

C23 $$\begin{aligned} \sum _y v y \int _0^T dt' \langle \Delta s(0), s(y,t') \rangle ^c = -v^2 T^2 \langle M,s(0,0)\rangle ^\textrm{c}. \end{aligned}$$

By expanding the connected correlation of differences and utilizing stationarity, the expression decomposes into a normal linear-response term and an irreversibility term:

C24 $$\begin{aligned} \mathfrak {L} = \liminf _{t\rightarrow \infty } (\mathfrak {L}_\textrm{norm}(t) - \mathfrak {L}_\textrm{irr}(t)) \end{aligned}$$

where:

C25 $$\begin{aligned} \mathfrak {L}_\textrm{norm}(t)&= \frac{1}{t} \sum _{x\in \mathbb {Z}} (x^2-(vt)^2) \langle s(x,t),s(0,0)\rangle ^c \end{aligned}$$

C26 $$\begin{aligned} \mathfrak {L}_\textrm{irr}(t)&= \frac{1}{2t} \sum _{x\in \mathbb {Z}} x^2\langle \tau _{t}\tau ^*_t( s(x)),s(0,0)\rangle ^c. \end{aligned}$$

## Footnotes

1 This is a standard result in $\hbox {C}^*$ algebras for finite range interaction quantum spin chains, or with exponentially decaying interactions, see [32, Theorem 6.2.11]

2 The constant $C_{A_1,\ldots ,A_n}$ depends linearly on the norms of the observables and polynomially on the sizes of their supports

3 Note that there is a difference of a factor of 2 with [13, Theorem 5.1], this is because in the present set-up the Onsager coefficient (49) is defined as an integral over t≥0 instead of ${\mathbb {R}}$

4 The result is irrespective of the site $x\in {\mathbb {Z}}$ of the density
